# A Canadian multicenter pediatric eosinophilic esophagitis cohort: Evidence for a nondilation approach to esophageal narrowing

**DOI:** 10.1002/jpr3.12149

**Published:** 2024-12-09

**Authors:** David Burnett, Vishal Avinashi, Thomas Hoang, Anthony Otley, Rabin Persad, Mary Sherlock, Hien Q. Huynh

**Affiliations:** ^1^ Dalhousie University Halifax Nova Scotia Canada; ^2^ University of Saskatchewan Saskatoon Saskatchewan Canada; ^3^ University of British Columbia Vancouver British Columbia Canada; ^4^ University of Alberta Edmonton Alberta Canada; ^5^ McMaster University Hamilton Ontario Canada

**Keywords:** dysphagia, stricture

## Abstract

**Objectives:**

Improving characterization of the narrowing phenotype in pediatric eosinophilic esophagitis (EoE).

**Methods:**

New pediatric EoE diagnoses from 2015 to 2018 were retrospectively identified in Vancouver (BC), Northern Alberta (AB), Hamilton (ON), and Nova Scotia (NS). Incidence rates were calculated using 2016 Federal census data. Clinical, endoscopic, and histologic data were gathered from diagnosis until the end of the follow‐up period (fall 2019).

**Results:**

The incidence of EoE in patients less than 15 years old was 5.4 per 100,000 person‐years. Of the 332 new diagnoses, 40 (12.0%) had endoscopically identified esophageal narrowing at diagnosis or during the follow‐up period, with a subset of 11 (27.5% of narrowed cohort) patients undergoing mechanical esophageal dilation. The median age at diagnosis and median duration of symptoms were higher in the cohort with narrowing than those without. Patient‐reported food bolus impaction and dysphagia were associated with esophageal narrowing. Trachealization was the endoscopic finding most commonly associated with esophageal narrowing. Of the 65 esophagogastroduodenoscopies performed in the follow‐up of a known esophageal narrowing, 4 of the 31 (13%) had resolution of this finding post mechanical dilation, and 19 of the 39 (49%) had resolution of the narrowing after initiation of new medical or dietary treatments (without dilation).

**Conclusions:**

EoE is common in Canadian children, with esophageal narrowing being present within a few years of diagnosis in 12% of cases. Interestingly, a large portion of narrowing resolved *without* mechanical dilation.

## Abstract (highlights)

**What is Known:**

- Reported incidence rates of pediatric eosinophilic esophagitis (EoE) vary widely between studies, as do the limited existing reports of rates of esophageal narrowing in pediatric EoE.
- Esophageal narrowing is considered a marker of severe EoE and is a cause of significant morbidity.
- Recent literature points to systemic steroids as an alternative to mechanical dilation for treating pediatric EoE esophageal narrowing.

**What is New:**

- In this multicenter retrospective Canadian study, esophageal narrowing was noted in 12% of patients. Most narrowing was present at diagnosis, with most others noted on endoscopy within 1–2 years of diagnosis.
- Nonsystemic/topical steroids and dietary‐elimination treatments were effective in resolving esophageal narrowing in many cases, without the need for systemic steroids or mechanical dilation.

## 1 INTRODUCTION

Eosinophilic esophagitis (EoE) is a chronic eosinophil‐predominant esophageal inflammatory condition affecting children and adults of all ages. Although EoE is one of the most common causes of dysphagia in pediatric patients, disease incidence in children remains poorly established, with a huge range reported between 0.7 and 13 cases per 100,000 person‐years.^1^, ^2^ Unfortunately, most of this literature used diagnostic criteria that did not account for proton pump inhibitor (PPI)‐responsive EoE, and so the accuracy of existing incidence rates is uncertain.

Esophageal narrowing remains the most worrisome complication of EoE. The narrowing phenotype has been well studied in the adult population, where a longer duration of untreated disease has led to narrowing in up to 70% of some cohorts.^3^ Esophageal narrowing is less common and less well characterized in pediatric EoE, with limited studies giving disparate rates of narrowing ranging from 0.2% to 28%.^4^, ^5^ While delay in diagnosis is a well‐established risk factor for EoE‐mediated esophageal narrowing in adults,^3^ no risk factors have been identified in pediatric cohorts. EoE‐mediated esophageal narrowing has typically been mechanically dilated in the past, with pediatric data mostly limited to small single‐center studies.^6^, ^7^, ^8^ There has been some interest in a nondilation approach to management of these cases, but to date this has been limited to a study demonstrating efficacy of systemic corticosteroids,^9^ and a single case report with dupilimab.^10^ The demonstration of a reversible quality (without mechanical dilation) to some pediatric EoE‐mediated esophageal narrowing contributes to mounting evidence that some cases of EoE‐associated esophageal narrowing in children are due to an *inflammatory* (rather than fibrotic) process.

The lack of research into the narrowing phenotype in pediatric EoE limits practitioners' ability to make evidence‐based treatment choices. Therefore, we present our multicenter retrospective chart review to describe the Canadian pediatric EoE experience, with a particular focus on the narrowing phenotype.

## 2 METHODS

### 2.1 Study design and case identification

We performed a retrospective chart review of new pediatric EoE diagnoses from 2015 to 2018 at four Canadian children's hospitals. The Stollery Children's Hospital in Edmonton, Alberta (AB) serves the northern half of the province (north of Red Deer, AB). The BCCH is located in Vancouver, British Columbia (BC) and is the only pediatric hospital in the province. The IWK Health Center is located in Halifax, Nova Scotia (NS) and is the only pediatric hospital in the province. McMaster Children's Hospital is in Hamilton, Ontario (ON) with a less well‐defined catchment area due to the presence of multiple children's hospitals in the province. All new diagnoses were identified at each site using a combination of prospectively kept patient registry (AB, BC, and ON), provincial histology database (AB, BC, and ON), hospital billing codes (ON and NS), and operating room database search (AB). All diagnoses were confirmed with individual chart review. All included patients met the diagnostic criteria outlined in the 2018 AGREE conference report,^11^ and were <16 years old at diagnosis.

The study was approved by the Research Ethics Board of all sites. Formal data‐sharing agreements were obtained between each site and the University of Alberta, the site of the centralized REDCap database server.

### 2.2 Disease incidence

The incidence of new EoE cases <15 years old in each region was calculated as described previously.^12^ The age limit of <15 years was set to align with the reporting of Canadian census data, and to reduce the risk of ascertainment bias with older teens who may be seen by adult gastroenterologists in some peripheral centers. Rural was defined as having a place of residence with a population of <10,000 on the most recent available census data. All new EoE diagnoses in Hamilton were included in our data set, but were excluded from the incidence calculations to avoid ascertainment bias due to its poorly defined catchment area.

### 2.3 Clinical, endoscopic and histology data

All data were retrospectively collected. All clinical decisions were at the discretion of the treating physician in discussion with patients and families, including but not limited to timing of endoscopies, number of biopsies, and all treatment decisions including timing/need for dilation. All cases were diagnosed between January 1, 2015 and December 31, 2018. The data collection period was extended until April 30, 2019, for a median follow‐up period of 808 days (interquartile range [IQR]: 466–1164 days), assuming no loss to follow‐up and discharge from pediatric gastroenterology on the 18th birthday.

Data were collected from endoscopy, pathology, and clinic reports at each center, and deidentified data were input into a centralized REDCap database. When histology eosinophil counts were recorded as “>*X*,” “*X*” was input as the eosinophil count. When endoscopic reference score (EREFS)^13^ was not recorded prospectively in endoscopy reports, the components of the score were extracted from the report at time of data entry. The presence and severity of narrowing were based on the endoscopist's subjective description as mild, moderate, or severe. If the severity of any endoscopy finding was not recorded, it was retrospectively assumed to be mild. If endoscopy findings were described as “moderate” in criteria that EREFS scoring only allows for mild or severe, then it was assumed to be mild.

Patients were placed into disease phenotype groupings based on their phenotype over either the entire study period (Table 2) or each individual scope (Table 3). The “Narrowing” group included patients who had an endoscopy with a focal esophageal narrowing. The “Narrowing with dilation” phenotype was a subgroup of the “Narrowing “ group, while the “Subtle signs of narrowing” group (such as creation of mucosal rent with passage of standard pediatric gastroscope) was identified as a stand‐alone phenotype. The “No narrowing” group included only patients without mention of either focal narrowing or the more subtle signs of narrowing in their endoscopy report(s).

### 2.4 Data analysis

Incidence rates are presented as cases per 100,000 person‐years, and were calculated in Microsoft Excel (2018, Microsoft Corp). Medians and odds ratios (ORs) are presented with IQRs, and were calculated using SPSS Statistics (Version 28; IBM Corp).

## 3 RESULTS

### 3.1 Disease incidence

Three hundred and thirty‐two new pediatric EoE cases were diagnosed at the four sites from 2015 to 2018. The incidence of EoE in patients <15 years old was calculated for AB, NS, and BC (Table 1). The combined incidence across all provinces was 5.4 cases per 100,000 person‐years, but with considerable variability between provinces.

**Table 1:** Incidence of EoE in patients less than 15 years old.

Age (years)	Northern Alberta			Nova Scotia			British Colombia			Combined
	Total	Rural	Urban	Total	Rural	Urban	Total	Rural	Urban	Total
0–4	6.4 (33)	0.9 (1)	8.0 (32)	1.8 (3)	1.2 (2)	0.6 (1)	1.8 (16)	0.3 (3)	1.5 (13)	3.3 (52)
5–9	9.4 (47)	6.0 (7)	10.4 (40)	9.8 (18)	3.3 (6)	6.5 (12)	2.4 (23)	0.3 (3)	2.1 (20)	5.4 (88)
10–14	12.1 (54)	5.5 (6)	14.2 (48)	7.6 (14)	6.0 (11)	1.6 (3)	5.1 (48)	0.3 (3)	4.7 (45)	7.1 (116)
0–14	9.1 (134)	4.1 (14)	10.6 (120)	6.5 (35)	3.5 (19)	3.0 (16)	3.1 (87)	0.3 (9)	2.8 (78)	5.4 (256)

*Note*: Incidence represented as incidence per 100,000 person‐years, with number of cases in brackets. Northern Alberta includes locations north of Red Deer, Alberta. Rural is defined as towns with populations <10,000.

Abbreviation: EoE, eosinophilic esophagitis.

### 3.2 Clinical, endoscopic, and histology data

Of the 332 new cases, the majority (281, 84.7% of the total cohort) had no signs of esophageal narrowing on any endoscopy during the follow‐up period (Table 2). Forty patients (12.0%) had one or more areas of focal esophageal narrowing on endoscopy at diagnosis or during the follow‐up period. Of the 40 patients with focal narrowing, 11 patients (11/332, 3.3%) underwent esophageal dilation. An additional 11 patients (3.3%) had more subtle endoscopic findings concerning narrowing, such as creating a rent with the passage of the endoscope.

**Table 2:** Clinical characteristics and endoscopic findings on diagnostic EGD for all study patients, categorized by disease phenotype observed over the follow‐up period.

	Narrowing	Narrowing with dilation (subset of Narrowing group)	Subtle signs narrowing (not a subset of Narrowing group)	No narrowing	Overall
Number of patients (%)	40 (12.0)	11 (3.3)	11 (3.3)	281 (84.7)	332
Clinical characteristics					
Atopic (%)	70.0%	63.6%	45.4%	72.2%	71.1%
Median age at diagnosis (years; IQR)	12.4 (8.9–14.1)	11.6 (3.4–13.7)	9.0 (5.4–13.9)	10.3 (6.1–13.6)	10.4 (6.3–13.7)
Median duration symptoms at diagnosis (years; IQR)	1.5 (0.5–3.0)	2.0 (1.1–3.8)	3.0 (1.5–5.0)	1.0 (0.5–2.8)	1.0 (0.5–3.0)
Median number of EGD/dilations during follow‐up period (IQR)	3.0 (2.0–4.8)	5.0 (4.0–7.0)	2.0 (2.0–3.0)	2.0 (2.0–3.0)	2.0 (2.0–3.0)
Symptoms at diagnosis (%)					
Food impaction	80.0	72.7	54.5	30.2	37.0
Dysphagia	82.5	72.7	63.6	58.4	61.4
Nausea/emesis	15.0	27.3	18.2	36.7	33.4
GER	7.5	0.0	9.1	16.0	14.8
Heartburn/chest pain	22.5	36.4	18.2	19.9	20.2
Abdo pain	2.5	0.0	27.3	28.8	25.6
Weight loss/FTT	5.0	0.0	0.0	15.3	13.6
Food refusal	0.0	0.0	0.0	1.8	1.5
Odynophagia	0.0	0.0	9.1	1.8	1.8
Globus sensation	0.0	0.0	9.1	0.7	0.9
Findings on diagnostic EGD (%)					
Linear furrowing	75.0	54.5	81.8	79.0	78.6
LOVP	27.5	36.4	72.7	37.0	37.0
Trachealization	50.0	36.4	27.3	14.2	19.0
White exudates	52.5	63.6	54.5	52.3	52.4
Narrowing	77.5	90.9	63.6	0.0	11.4

*Note*: Narrowing with dilation is a subset of the Narrowing clinical cohort who underwent mechanical dilation; the other cohorts (subtle signs narrowing, No narrowing) are separate nonoverlapping cohorts.

Abbreviations: EGD, esophagogastroduodenoscopy; FTT, failure to thrive; GER, gastroesophageal reflux; IQR, interquartile range; LOVP, loss of vascular pattern.

The median age at diagnosis was higher in those who developed narrowing (either present at diagnosis or developed during follow‐up) than those who did not, but there was significant overlap of IQRs (Figure S1). Similarly, the median duration of symptoms before diagnosis was higher in the narrowed cohort (1.5 years; IQR: 0.5–3.0) and highest in those who underwent dilation (2.0 years; IQR: 1.1–3.8), but again with large variation (Figure S2). The median duration of symptoms before endoscopic confirmation of an esophageal narrowing was 3 years (IQR: 1.06–5.50).

The rate of patient‐reported food‐impaction (80% vs. 30.2%; OR: 8.8, 95% confidence interval [CI]: 3.9–19.9) and dysphagia (82% vs. 58.4%; OR: 3.3, 95% CI: 1.3–7.8) at diagnosis were higher in patients who went on to have esophageal narrowing, while other symptoms were less discriminatory (Table 2). 77.5% of those who had a narrowing during the study period had this finding on diagnostic endoscopy, while 90.9% of those who went on to undergo mechanical dilation had focal narrowing at diagnosis. On diagnostic endoscopy, trachealization had the highest specificity for those who went on to have esophageal narrowing (50.0%) compared with those who never had narrowing (14.2%; OR: 5.7, 95% CI: 2.8–11.5).

When the endoscopic data were categorized by findings at *each* endoscopy (Table 3), this pattern was maintained, with the exception that trachealization was *least* likely to be documented on endoscopies involving dilation. The median EREFS score and peak eosinophil count were lowest in endoscopies involving esophageal dilation, although with wide variability.

**Table 3:** Endoscopic findings on diagnostic and follow‐up EGD for all study patients, categorized by disease phenotype observed during each scope.

	Narrowing	Narrowing with dilation (subset of Narrowing group)	Subtle signs narrowing	No narrowing	Overall
Number of endoscopies	72	27	13	795	880
Findings on EGD (% scopes)					
Linear furrowing	63.9	40.7	53.8	67.4	67.0
LOVP	34.7	44.4	53.8	37.1	37.3
Trachealization	34.7	3.7	23.1	12.1	14.1
White exudates	43.1	29.6	46.2	41.5	41.8
Narrowing	100.0	100.0	100.0	0.0	10.8
EREFS score (median, IQR)	3 (1–4)	1 (1–3)	3 (2–5)	2 (1–3)	2 (1–3)
Peak eosinophil count (median, IQR)	30 (11–60)	13 (3–27)	25 (17–85)	40 (16–67)	38 (15–67)

*Note*: Narrowing with dilation is a subset of the Narrowing clinical cohort who underwent mechanical dilation; the other cohorts (subtle signs narrowing, No narrowing) are separate nonoverlapping cohorts. Peak eosinophil count reported as eosinophils per high‐powered field.

Abbreviations: EGD, esophagogastroduodenoscopy; EREFS, endoscopic reference score; IQR, interquartile range; LOVP, loss of vascular pattern.

The overall cohort underwent 880 endoscopies during the study period, including 72 endoscopies with the finding of focal esophageal narrowing. A subgroup of 11 patients with focal narrowing underwent 27 endoscopic esophageal dilations, and an additional 10 esophageal dilations by interventional radiology (IR). The majority (31, 77%) of esophageal narrowing was noted on diagnostic endoscopy, with the remaining areas of narrowing first noted a median of 345 days (IQR: 252–997) after diagnosis. The location of focal narrowing was distributed between the proximal, mid, and distal esophagus at 21 (25.6%), 29 (35.4%), and 39 (47.6%) endoscopies, respectively. This includes 13 (15.9%) endoscopies reporting focal narrowing in multiple regions but excludes 2 (2.4%) where no location was specified. Severity of narrowing was not documented in 9.8% of endoscopies/IR dilations, but in the remainder was subjectively reported as mild, moderate, or severe (32.9%, 52.4%, and 4.9%, respectively). Of the 11 patients who underwent dilation, the median number of dilations during the follow‐up period was 3 (IQR: 1–5), with a median of 81 days (IQR: 49–148) between dilations. The majority (81.5%) of the 27 endoscopic dilations were done using pneumatic balloon dilation, with the remainder done via bougie. The median increase in lumen diameter from dilation was 2.0 mm (IQR: 1.8–3.0) for a final median diameter of 12.0 mm (IQR: 10.0–14.0).

### 3.3 Treatment efficacy

A subset of 65 follow‐up endoscopies was assessed, which were done *after* a procedure (endoscopy, endoscopic dilation, or IR‐dilation) documented focal esophageal narrowing (Table 4). Narrowing was no longer observed in 24 (36.9%) and the narrowing was therefore considered resolved. The patients with resolved narrowing included 1 where no new treatment had been introduced, 4 where mechanical dilation had been performed, and 19 where new medical or dietary therapy had been introduced. There was some overlap between these groups, with some patients receiving dilation and then starting a new medical/dietary treatment in the interval. Of those started on an individual new medical therapy (not a combination of therapies, and not mechanically dilated), the rates of resolution of narrowing on follow‐up endoscopy were generally high, including 1 out of 1 (100%) on systemic steroids, 7 out of 13 (54%) on topical steroids, 3 out of 4 (75%) on dietary therapy, and 4 out of 12 (33%) on PPI therapy. There was no obvious difference between the median peak eosinophil count or median EREFS score between those patients with narrowing which resolved post‐dilation versus post‐medical management.

**Table 4:** Efficacy of interval treatment in patients with resolved focal narrowing on follow‐up EGD.

	Narrowing resolved on follow‐up EGD (*N*)	Peak Eo count (median, IQR)	EGD with peak Eo count <15/HPF (*N*)	EREFS (median, IQR)	EGD with EREFS of 0 (*N*)
No new treatment	1 out of 1 follow‐up EGDs	100 (100)	0	2 (2)	0
Post‐dilation	4 out of 31 follow‐up EGDs	28.5 (1.8–57.5)	2	2 (0–4)	2
New medical/dietary treatments	19 out of 39 follow‐up EGDs	32.0 (0–80.0)	7	2 (1–2)	4

*Note*: Peak Eo count, Eo count < 15/HPF, EREFS, and EREFS of 0 are taken only from follow‐up EGDs which showed resolution of previously documented focal narrowing.

Abbreviations: EGD, esophagogastroduodenoscopy; Eo, eosinophil; EREFS, endoscopic reference score; HPF, high‐powered field; IQR, interquartile range.

Additional information on the effects of medical and dietary therapies is included in Table S1. Given the limitations of this data (particularly the lack of adherence data, and the number of patients started on two or more therapies concurrently), no statistical analysis was performed.

## 4 DISCUSSION

We present the first multicenter Canadian pediatric EoE data that uses the 2018 AGREE diagnostic criteria,^11^ with a cumulative incidence of 5.4 per 100,000 person‐years (Table 1). This incidence is within the extremely variable incidence rates reported in previous studies. Although the stratification of census data required limiting our incidence calculations to the less than 15‐year‐old age group, this only serves to further reduce the potential for ascertainment bias due to adolescent patients diagnosed by adult practitioners.

Our multicenter cohort is the largest described to date, with detailed data on esophageal narrowing in pediatric EoE. Of the 332 new diagnoses, 12.0% of patients developed focal esophageal narrowing during our 4‐year follow‐up period. Interestingly, the majority (77%) of these areas of narrowing were noted at diagnostic endoscopy, with the remainder noted not long after (median: 345 days, IQR: 252–997). The rates of pediatric EoE‐mediated esophageal narrowing in the literature are incredibly varied, from as low as 0.2% to as high as 28%.^4^, ^5^ It remains unclear whether this variability represents differences in disease severity, unique phenotypes, or due to study confounders such as differences in practice patterns, and unequitable access to subspecialist care. In the context of this variability, our study's strong case identification processes will make our narrowing rate an important touchstone moving forward.

There are several important trends in the endoscopic and histologic data presented here. Most notably, patient‐reported history of food bolus impaction and trachealization on diagnostic esophagogastroduodenoscopy (EGD) were the most common in those who went on to have esophageal narrowing (Table 2). Interestingly, when follow‐up and interventional endoscopies were included (Table 3), this trend became less clear, and the trend was lost completely in those undergoing endoscopic dilation. Reduced rates of trachealization and lower overall EREFS scores in those undergoing dilation could relate to incomplete documentation of endoscopic findings for interventional procedures, or could point to a theoretically increased therapy adherence at the time of mechanical dilation (which would be supported by the reduced peak eosinophil count in this group) due to presumed increased symptom severity associated with an area of narrowing.

Esophageal narrowing was fairly evenly spaced throughout the esophagus, as would be expected from a panesophageal process. The rate of focal narrowing being documented at multiple esophageal levels (15.9%) is similar to what has been reported previously in children.^9^ Although the majority of our narrowing was reported as only mild or moderately severe, we must acknowledge that these terms were not defined prospectively, and in many cases, the severity was not documented in endoscopy reports.

The mechanical dilations described are a mixture of endoscopic balloon, endoscopic bougie, and IR dilations, as has been described elsewhere. The median increase in lumen diameter post‐dilation of 2 mm is in line with standard practice. While a number of follow‐up scopes showed resolution of esophageal narrowing post‐dilation, it should be noted that each dilated patient underwent a median of 3 dilations (IQR: 1–5), and were all concurrently on other EoE‐therapies. Additionally, of the two patients with severe narrowing who underwent mechanical dilation, there was no follow‐up endoscopy that documented the resolution of narrowing during our study period. Information regarding safety outcomes from mechanical dilation in the AB cohort has been described previously.^12^

Our most surprising result was the high number of cases of esophageal narrowing that resolved on follow‐up scope after initiating medical/dietary therapy, without need for mechanical dilation (Table 4). While a recent study has described efficacious treatment of narrowing with systemic steroids,^9^ ours is the first to document resolution with PPI, topical steroids, and dietary exclusions. While these results must be interpreted with some caution, given the retrospective nature of our study and the lack of standardized protocol to determine the need for follow‐up endoscopy, the results are nonetheless quite striking. The data support the proposal that many pediatric EoE‐mediated areas of esophageal narrowing are inflammatory (rather than fibrostenotic), but could also fit with the more controversial position that EoE‐mediated fibrosis is a reversable phenomenon.^14^ Additional evidence of an inflammatory process driving the esophageal narrowing is the uniformity between subgroups in the duration of symptoms at diagnosis and the age at diagnosis (Figures S1 and S2). This runs contrary to the well‐established escalating rates of esophageal narrowing seen in adults with disease progression.^3^, ^15^ Finally, the short duration of symptoms in many of our cases before establishing an area of narrowing (median 3 years; IQR: 1.06–5.50) would also point away from a slowly progressive fibrotic process. Regardless of underlying pathophysiology of EoE‐mediated esophageal narrowing, our data support studies which suggest that medication and/or dietary therapies should be trialed before proceeding to mechanical dilation.^9^, ^10^

Additional details about the efficacy of the medication and dietary treatments are provided for the sake of completeness (Table S1). While these data provide valuable insight into “real world” clinical practice, it should be interpreted with caution given the lack of compliance data and its retrospective collection.

There are a few important limitations of our study. First, the retrospective interpretation of subjective endoscopy and clinic reports imparts a risk of bias. This is most problematic with the endoscopists' assessment and description of esophageal narrowing, which also affects clinicians' perceived “need” for treatments (including dilations). Without a secondary endoscopic technology to quantify the lumen diameter and distensibility, such as endoluminal functional lumen imaging probe (EndoFLIP) (which remains unavailable at many pediatric centers, and its role in EoE management is still not fully defined), such assessments will remain inherently subjective. However, in follow‐up prospectively designed studies, there will also be an opportunity to use semi‐objective narrowing criteria that have been used elsewhere.^16^ Second, while all cases met the 2018 AGREE diagnostic criteria for EoE, there may have been some cases of PPI‐responsive EoE that were not offered endoscopy, given the cases were identified before publication of these criteria. Thirdly, the treatments described may vary based on individual physician practice patterns, rather than an intrinsic quality of the patient's disease phenotype. Additionally, the lack of symptom data and treatment details (including duration, dosage, and adherence) limits the conclusions we can draw. Finally, although the histology results were not central to our results, we must acknowledge that the inclusion of cell‐count values that had been reported as “greater than” a value does limit the accuracy of the histology data.

A few strengths of this work are worth highlighting. First, our case‐finding strategies yield highly reliable incidence rates. Second, the size and multicenter nature of the study help increase the generalizability of our results. Finally, collecting data on the nonnarrowed cases has allowed an important element of comparison between disease phenotypes.

In conclusion, we show that pediatric EoE is common in Canadian children, but with variable rates within the country's provinces. We also demonstrate that esophageal narrowing is a common complication in this condition, but that this can often be treated with medical and dietary therapies.

More work must be done to understand the pathophysiology of pediatric EoE‐mediated esophageal narrowing, with particular emphasis on anything that may differentiate adult and pediatric phenotypes. This will help us move toward more evidence‐based treatment approaches. Until such data exist, we advocate for attempting medical therapy before resorting to mechanical dilation for all esophageal narrowing in cases of pediatric EoE.

## CONFLICTS OF INTEREST STATEMENT

Dr. Avinashi was part of an advisory committee for Avir Pharma in 2021. Dr. Sherlock has been an Advisory Board Member for Sanofi and has received a speaker fee from Janssen. Dr. Burnett has received a speaker fee from Sanofi. Dr. Huynh has been an Advisory Board Member for Sanofi and receved a speaker fee for Sanofi as as well Janssen.

## Supporting information

Supporting information.

Supporting information.
