# Supplementary material for: A Canadian multicenter pediatric eosinophilic esophagitis cohort: Evidence for a nondilation approach to esophageal narrowing
Source: JPGN Rep. 2024 Dec 9;6(1):19–26. doi: 10.1002/jpr3.12149 (PMC11810807; doi:10.1002/jpr3.12149)

Supplementary Figure 1. Median age at diagnosis for all study patients, categorized by disease phenotype observed over follow-up period


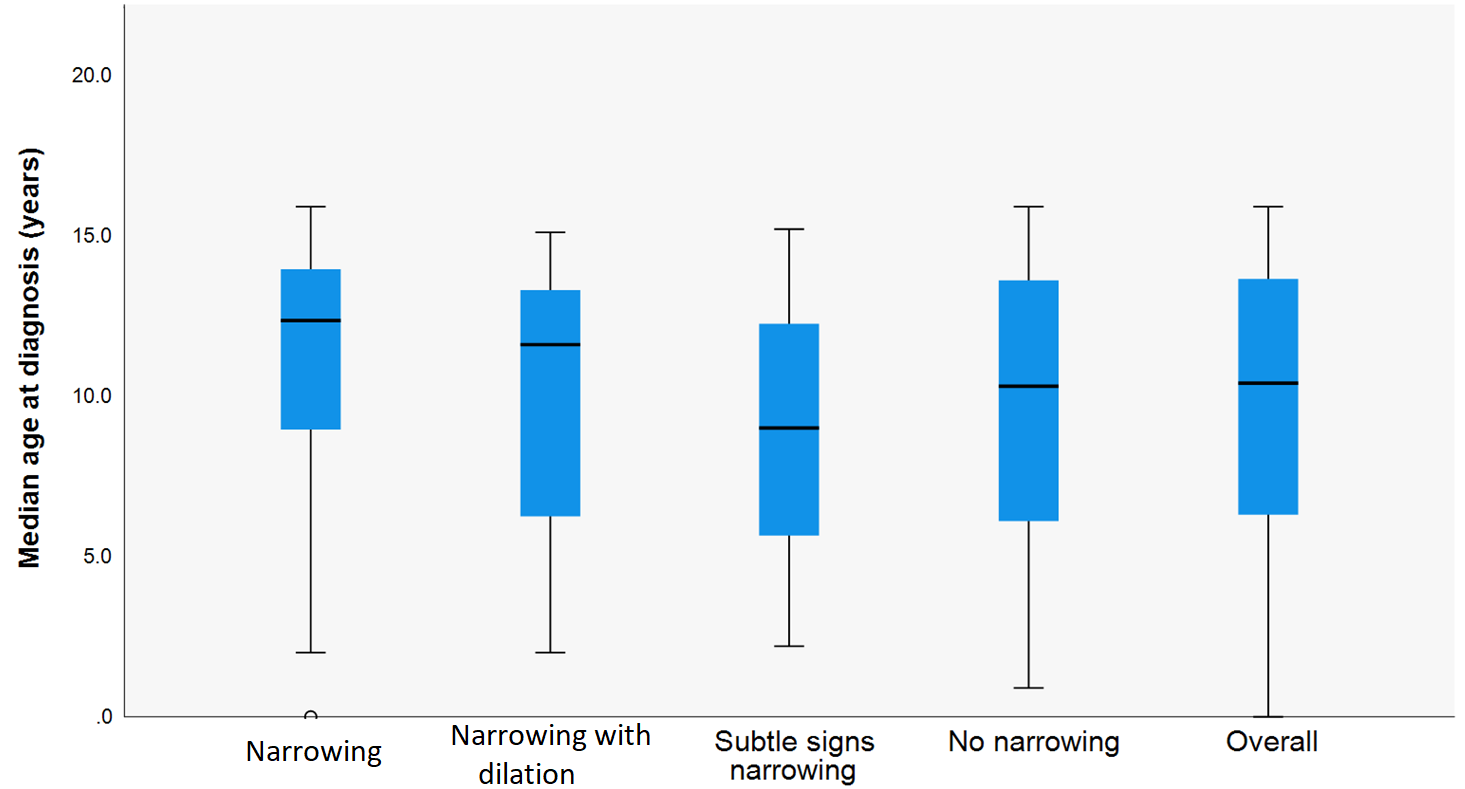


Supplementary Figure 2. Median duration of symptoms at diagnosis for all study patients, categorized by disease phenotype observed over follow-up period


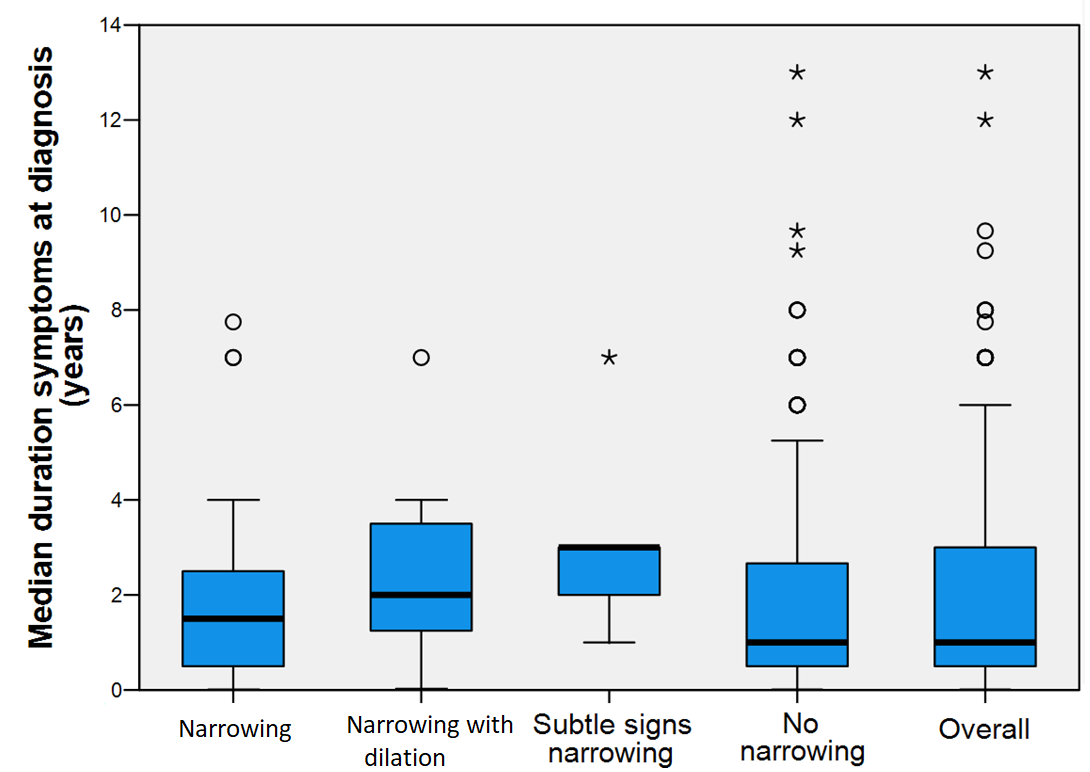

Supplement: Supplementary file 1 — Supporting information. [file JPR3-6-19-s002.docx]
