# Supplementary material for: A Canadian multicenter pediatric eosinophilic esophagitis cohort: Evidence for a nondilation approach to esophageal narrowing
Source: JPGN Rep. 2024 Dec 9;6(1):19–26. doi: 10.1002/jpr3.12149 (PMC11810807; doi:10.1002/jpr3.12149)
Supplement: Supplementary file 2 — Supporting information. [file JPR3-6-19-s001.docx]

Table 1. Incidence of EoE in patients less than 15 years old

| Age (years) | Northern Alberta | | | Nova Scotia | | | British Colombia | | | Combined |
| --- | --- | --- | --- | --- | --- | --- | --- | --- | --- | --- |
|  | Total | Rural | Urban | Total | Rural | Urban | Total | Rural | Urban | Total |
| 0 - 4 | 6.4 (33) | 0.9 (1) | 8.0 (32) | 1.8 (3) | 1.2 (2) | 0.6 (1) | 1.8 (16) | 0.3 (3) | 1.5 (13) | 3.3 (52) |
| 5 - 9 | 9.4 (47) | 6.0 (7) | 10.4 (40) | 9.8 (18) | 3.3 (6) | 6.5 (12) | 2.4 (23) | 0.3 (3) | 2.1 (20) | 5.4 (88) |
| 10 - 14 | 12.1 (54) | 5.5 (6) | 14.2 (48) | 7.6 (14) | 6.0 (11) | 1.6 (3) | 5.1 (48) | 0.3 (3) | 4.7 (45) | 7.1 (116) |
| 0 - 14 | 9.1 (134) | 4.1 (14) | 10.6 (120) | 6.5 (35) | 3.5 (19) | 3.0 (16) | 3.1 (87) | 0.3 (9) | 2.8 (78) | 5.4 (256) |

Incidence represented as incidence per 100,000 person-years, with number of cases in brackets. Northern Alberta includes locations north of Red Deer, Alberta. Rural is defined as towns with populations < 10,000. EoE (Eosinophilic esophagitis).

Table 2. Clinical characteristics and endoscopic findings on diagnostic EGD for all study patients, categorized by disease phenotype observed over follow-up period

|  | Narrowing | Narrowing with dilation  *[subset of*  *Narrowing group]* | Subtle signs narrowing  [*not* a subset of  Narrowing group] | No narrowing | Overall |
| --- | --- | --- | --- | --- | --- |
| Number of patients (%) | 40 (12.0) | 11 (3.3) | 11 (3.3) | 281 (84.7) | 332 |
| *Clinical characteristics:* |  |  |  |  |  |
| Atopic (%) | 70.0% | 63.6% | 45.4% | 72.2% | 71.1% |
| Median age at diagnosis (years; IQR) | 12.4 (8.9 - 14.1) | 11.6 (3.4 - 13.7) | 9.0 (5.4 - 13.9) | 10.3 (6.1 - 13.6) | 10.4 (6.3 - 13.7) |
| Median duration symptoms at diagnosis (years; IQR) | 1.5 (0.5 - 3.0) | 2.0 (1.1 - 3.8) | 3.0 (1.5 - 5.0) | 1.0 (0.5 - 2.8) | 1.0 (0.5 - 3.0) |
| Median number of EGD/dilations during follow-up period (IQR) | 3.0 (2.0 - 4.8) | 5.0 (4.0 - 7.0) | 2.0 (2.0 - 3.0) | 2.0 (2.0 - 3.0) | 2.0 (2.0 - 3.0) |
| *Symptoms at diagnosis (%)* |  |  |  |  |  |
| Food impaction | 80.0 | 72.7 | 54.5 | 30.2 | 37.0 |
| Dysphagia | 82.5 | 72.7 | 63.6 | 58.4 | 61.4 |
| Nausea/Emesis | 15.0 | 27.3 | 18.2 | 36.7 | 33.4 |
| GER | 7.5 | 0.0 | 9.1 | 16.0 | 14.8 |
| Heartburn/Chest pain | 22.5 | 36.4 | 18.2 | 19.9 | 20.2 |
| Abdo pain | 2.5 | 0.0 | 27.3 | 28.8 | 25.6 |
| Weight loss/FTT | 5.0 | 0.0 | 0.0 | 15.3 | 13.6 |
| Food refusal | 0.0 | 0.0 | 0.0 | 1.8 | 1.5 |
| Odynophagia | 0.0 | 0.0 | 9.1 | 1.8 | 1.8 |
| Globus sensation | 0.0 | 0.0 | 9.1 | 0.7 | 0.9 |
| *Findings on diagnostic EGD (%)* | |  |  |  |  |
| Linear furrowing | 75.0 | 54.5 | 81.8 | 79.0 | 78.6 |
| LOVP | 27.5 | 36.4 | 72.7 | 37.0 | 37.0 |
| Trachealization | 50.0 | 36.4 | 27.3 | 14.2 | 19.0 |
| White exudates | 52.5 | 63.6 | 54.5 | 52.3 | 52.4 |
| Narrowing | 77.5 | 90.9 | 63.6 | 0.0 | 11.4 |

Narrowing with dilation is a subset of the Narrowing clinical cohort who underwent mechanical dilation; the other cohorts (Subtle signs narrowing, No narrowing) are separate non-overlapping cohorts. EGD (esophagogastroduodenoscopy). IQR (interquartile range). GER (gastroesophageal reflux). FTT (failure to thrive). LOVP (loss of vascular pattern).

Table 3. Endoscopic findings on diagnostic and follow-up EGD for all study patients, categorized by disease phenotype observed during each scope

|  | Narrowing | Narrowing with dilation  *[subset of*  *Narrowing group]* | Subtle signs narrowing | No narrowing | Overall |
| --- | --- | --- | --- | --- | --- |
| *Number of endoscopies* | 72 | 27 | 13 | 795 | 880 |
| *Findings on EGD (% scopes)* | |  |  |  |  |
| Linear furrowing | 63.9 | 40.7 | 53.8 | 67.4 | 67.0 |
| LOVP | 34.7 | 44.4 | 53.8 | 37.1 | 37.3 |
| Trachealization | 34.7 | 3.7 | 23.1 | 12.1 | 14.1 |
| White exudates | 43.1 | 29.6 | 46.2 | 41.5 | 41.8 |
| Narrowing | 100.0 | 100.0 | 100.0 | 0.0 | 10.8 |
| EREFS score (median, IQR) | 3 (1 - 4) | 1 (1 - 3) | 3 (2 - 5) | 2 (1 - 3) | 2 (1 - 3) |
| Peak eosinophil count (median, IQR) | 30 (11 - 60) | 13 (3 - 27) | 25 (17 - 85) | 40 (16 - 67) | 38 (15 - 67) |
|  |  |  |  |  |  |

Narrowing with dilation is a subset of the Narrowing clinical cohort who underwent mechanical dilation; the other cohorts (Subtle signs narrowing, No narrowing) are separate non-overlapping cohorts. EGD (esophagogastroduodenoscopy). IQR (interquartile range). LOVP (loss of vascular pattern). EREFS (Endoscopic reference score). Peak eosinophil count reported as eosinophils per high powered field.

Table 4. Efficacy of interval treatment in patients with resolved focal narrowing on follow-up EGD

|  | Narrowing resolved on follow-up EGD (N) | Peak Eo count  (median, IQR) | EGD with peak Eo count < 15/HPF (N) | EREFS (median, IQR) | EGD with EREFS of 0 (N) |
| --- | --- | --- | --- | --- | --- |
| No new treatment | 1 of 1 follow-up EGDs | 100 (100) | 0 | 2 (2) | 0 |
| Post-dilation | 4 of 31 follow-up EGDs | 28.5 (1.8 – 57.5) | 2 | 2 (0 – 4) | 2 |
| New medical/dietary treatments | 19 of 39 follow-up EGDs | 32.0 (0 – 80.0) | 7 | 2 (1-2) | 4 |

*EGD (esophagogastroduodenoscopy). Eo (Eosinophil). IQR (interquartile range).* *HPF (high powered field). EREFS (Endoscopic reference score). Peak Eo count, Eo count < 15/HPF, EREFS and EREFS of 0 are taken only from follow-up EGDs which showed resolution of previously documented focal narrowing.*
